# Relationship between Sustained Reductions in Plasma Lipid and Lipoprotein Concentrations with Apheresis and Plasma Levels and mRNA Expression of PTX3 and Plasma Levels of hsCRP in Patients with HyperLp(a)lipoproteinemia

**DOI:** 10.1155/2016/4739512

**Published:** 2016-01-19

**Authors:** Claudia Stefanutti, Fabio Mazza, Michael Steiner, Gerald F. Watts, Joel De Nève, Daniela Pasqualetti, Juergen Paal

**Affiliations:** ^1^Extracorporeal Therapeutic Techniques Unit, Lipid Clinic and Atherosclerosis Prevention Centre, Immunohematology and Transfusion Medicine, Department of Molecular Medicine, “Sapienza” University of Rome, “Umberto I” Hospital, Viale del Policlinico, 00161 Rome, Italy; ^2^Medizinisches Labor Rostock, Rostock, Germany; ^3^Cardiovascular Medicine, Royal Perth Hospital, School of Medicine and Pharmacology, University of Western Australia, Australia; ^4^LabOmics S.A., Nivelles, Belgium; ^5^Fresenius Medical Care Deutschland GmbH, Bad Homburg, Germany

## Abstract

The effect of lipoprotein apheresis (Direct Adsorption of Lipids, DALI) (LA) on plasma levels of pentraxin 3 (PTX3), an inflammatory marker that reflects coronary plaque vulnerability, and expression of PTX3 mRNA was evaluated in patients with hyperLp(a)lipoproteinemia and angiographically defined atherosclerosis/coronary artery disease. Eleven patients, aged 55 ± 9.3 years (mean ± SD), were enrolled in the study. PTX3 soluble protein levels in plasma were unchanged by 2 sessions of LA; however, a downregulation of mRNA expression for PTX3 was observed, starting with the first session of LA (*p* < 0.001). The observed reduction was progressively increased in the interval between the first and second LA sessions to achieve a maximum decrease by the end of the second session. A statistically significantly greater treatment-effect correlation was observed in patients undergoing weekly treatments, compared with those undergoing treatment every 15 days. A progressive reduction in plasma levels of C-reactive protein was also seen from the first session of LA, with a statistically significant linear correlation for treatment-effect in the change in plasma levels of this established inflammatory marker (*R* ^2^ = 0.99; *p* < 0.001). Our findings suggest that LA has anti-inflammatory and endothelium protective effects beyond its well-established efficacy in lowering apoB100-containing lipoproteins.

## 1. Introduction

The development and progression of atherosclerosis involves the immune response and inflammation. Among the major markers of inflammation are two proteins of the pentraxin superfamily: the acute phase proteins C-reactive protein (CRP) and pentraxin 3 (PTX3). The pentraxins are acute phase proteins comprising five units in a cyclic pentameric structure held together by noncovalent bonds. All pentraxins share a characteristic C-terminal domain (pentraxin domain) of 200 amino acids that has within it 8 amino acids that are highly conserved (pentraxin signature) and are structurally different according to their short and long chain.

Short pentraxins found in humans include the serum amyloid P component (SAP) and CRP [1]. Among the long pentraxins, PTX3 or tumor necrosis factor- (TNF-) inducible gene 14 protein (TGS-14) was the first to be described at the beginning of the 1990s, when a new domain was identified as an interleukin-1 (IL-1) inducible gene in endothelial cells and a TNF-inducible gene in fibroblasts [2, 3]. PTX3 is comprised of a C-terminus of 203 amino acids (the domain that is shared with all pentraxins) and a characteristic N-terminus of 178 amino acids that makes it a long pentraxin. The role of PTX3 in inflammatory processes has been confirmed by* in vitro* studies on cultures of smooth muscle cells from healthy human arteries incubated with modified atherogenic lipoproteins [4].* In vivo* studies have found a relationship between the cells of carotid atherosclerotic plaques and myocytes of patients with acute myocardial infarction with high levels of PTX3 [5–7]. In contrast to CRP, PTX3 is expressed at very low levels in the liver and is not expressed constitutively by the central nervous system if not induced by inflammatory stimuli. The different sites of tissue expression reflect the different, heterogeneous roles. Levels of PTX3 are elevated in patients with cardiovascular disease (CVD) and high values in the general population are considered to be predictive of CVD, especially in patients with arterial hypertension and with dyslipidemia. Recent studies suggest that PTX3 may represent a novel effective marker of cardiovascular risk [6–8]. Lipoprotein apheresis (LA) with the DALI (Direct Adsorption of Lipids) system is a relatively new technique that offers greater cardiovascular protection through the selective removal of low-density lipoprotein, very low-density lipoprotein, lipoprotein(a) (Lp(a)), and inflammatory cytokines directly from whole blood [9]. With its filtering action, extracorporeal LA plays an important role in reducing the progression of coronary artery disease (CAD) in subjects with familial hypercholesterolemia (FH) and hyperLp(a)lipoproteinemia (HyperLp(a)) [10, 11]. Currently there is no reported evidence of the effects of LA on plasma levels of soluble PTX3 and on PTX3 messenger ribonucleic acid (mRNA) expression. The aim of the current study was to evaluate the effects of LA on plasma levels and mRNA expression of PTX3, throughout two consecutive (weekly or biweekly) treatment sessions of LA, in patients with genetically determined dyslipidemia (HyperLp(a)) and associated CAD, assessed by aortocoronary catheterization.

## 2. Materials and Methods

### 2.1. Patients

Eleven patients (10 males and 1 female), aged 55 ± 9.3 years (mean ± standard deviation [SD]), were enrolled in the study. All patients were affected by HyperLp(a). Ten patients had documented CAD, and 1 had nonstenotic atherosclerotic wall abnormalities assessed by aortocoronary catheterization (Table 1). All patients were treated with LA. The frequency of treatment was weekly in 6 patients and biweekly (every 15 days) in 5 patients.

### 2.2. Ethics

Written informed consent was obtained from all patients, according to the recommendations of the Declaration of Helsinki, guiding physicians in biomedical research involving human subjects (adopted by the 18th World Medical Association General Assembly, Helsinki, Finland, June 1964, and amended by the 29th World Medical Assembly, Tokyo, Japan, October 1975, the 35th World Medical Assembly, Venice, Italy, October 1983, and the 41st World Medical Assembly, Hong Kong, September 1989).

### 2.3. DALI Procedure

The DALI system is based on the binding of positively charged apolipoprotein B100 to negatively charged polyacrylic acid covalently bound to polymethacrylamide. In the present study, DALI apheresis procedures were performed weekly, with all patients subjected to the same standardized protocol (adsorber volume 500 mL and/or 750 mL gel; flow rate Qb 60 mL/min). Anticoagulation was achieved using an initial bolus dose of heparin 20 IU/kg, followed by infused citric acid 1 mL/20–40 mL blood. Vascular access was via the antecubital veins, and the mean volume processed per session was ~10000 mL.

### 2.4. Laboratory Activities

#### 2.4.1. Sample Collection

Four samples from each of the 11 patients were collected and stored. The 4 samples for each patient correspond to the following: blood collected before first apheresis treatment (considered as baseline), PRE1; blood collected after first apheresis treatment, POST1; blood collected before second apheresis treatment, PRE2; blood collected after second apheresis treatment, POST2.

Two naïve samples (one from a nonsick patient and one from a sick patient) were collected and processed under the same procedure, for downstream treatment and analysis procedures adjustments and validation. After collection was completed, the samples were shipped under dry ice to LabOmics S.A. (Nivelles, Belgium) for proteomics and transcriptomics analysis.

#### 2.4.2. RT-qPCR Analysis

*(A) RNA Extraction.* A total of 42 RNA falcon tubes were shipped to LabOmics including two tubes ABN and NORM for evaluation purpose. We used TRIzol extraction procedure followed by cleanup method using the QIAGEN RNeasy Mini Kit (http://www.qiagen.com/). The RNA was eluted in 50 *μ*L water (RNAse free). RNA concentrations and the ratio of OD at 260 to 280 nm (A260/280) were measured by NanoDrop 1000 Spectrophotometer (Thermo Scientific) and the RNA quality was controlled by Agilent 2100 Bioanalyzer microfluidic electrophoresis chips. Each RNA sample was stored at −80°C.

*(B) cDNA Synthesis.* RevertAid H Minus First-Strand cDNA Synthesis Kit (Thermo Scientific) was used to retrotranscribe 250 ng of RNA into cDNA. The kit is supplied with both oligo(dT)18 and random hexamer primers. The oligo(dT)18 primer anneals selectively to the poly(A) tail of mRNA. Random hexamer primers do not require the presence of poly(A). We evaluated the performance of use of the oligo(dT)18 primers over random hexamer primers. Reverse transcription of mRNA extracted from 4 patients was investigated and quantitative real-time polymerase chain reaction (qPCR) analyses were performed in triplicate using GAPDH primers in the presence of oligo(dT) and random hexamers. The yield of cDNA was investigated and showed no major difference between random hexamer and oligo(dT)18 primers. This result was confirmed by evaluating the cDNA synthesis efficiency using qPCR.

*(C) qPCR Reactions.* The SYBR Green PCR Master Mix is supplied in a 2x concentration (Life Technologies PN 4309155). The mix is optimized for SYBR Green reactions and contains SYBR Green I Dye, AmpliTaq Gold DNA Polymerase, dNTPs with dUTP, Passive Reference, and optimized buffer components. Real-time PCR was conducted using an ABI 7900HT Sequence Detection System (Applied Biosystems, Foster City, California, USA) and quantification was accomplished with the accompanying software package software version SDS 2.4. The PCR reaction was carried out in 25 *μ*L volumes and performed in MicroAmp Optical 96-well Reaction Plate Life Technologies (PN N801-0560) and MicroAmp Optical Adhesive Film Kit Life Technologies (PN 4313663). All genes for each sample were assayed in triplicate in the presence of 2 *μ*L of 40-fold diluted first-strand cDNA synthesis reaction mixture and 20 picomoles of primers. Polymerase activation at 95°C for 10 min was followed by 40 cycles of 15 s at 95°C, 30 s at 60°C, and 30 s at 60°C. The dissociation curve analysis, which evaluates each PCR product to be amplified from single cDNA, was carried out in accordance with the manufacturer's protocol. Expression levels were reported as cycle threshold (Ct) values. Because it was envisioned that ACT1 would serve as a single-gene normalization control, this gene was included on each plate. Raw qPCR expression measures were quantified using Applied Biosystems SDS software and reported as Ct values. The Ct value represents the number of cycles or rounds of amplification required for the fluorescence of a gene or primer pair to surpass an arbitrary threshold. The magnitude of the Ct value is inversely proportional to the expression level so that a gene expressed at a high level will have a low Ct value and vice versa. Triplicate Ct values were combined by averaging and standard deviation was calculated. Data normalization was carried out against ACT1 gene an endogenous unregulated reference gene transcript.

The SYBR Green assay was capable of detecting ≥0.01 pg of PTX3 mRNA ≥0.1 ng of total RNA with high specificity and reproducibility (coefficient of variation for delta Ct <0.9%).

#### 2.4.3. Proteomics: Enzyme-Linked Immunosorbent Assay (ELISA)

The ELISA was performed using a commercial ELISA kit purchased from Cusabio (http://www.cusabio.com/) and according to manufacturer's instructions. PTX3 detection range was 0.156 ng/mL–10 ng/mL and limit of sensitivity 0.039 ng/mL. The PTX3 ELISA showed no significant cross-reactivity or interference between human PTX3 and analogues. Measurements of clinical samples were done in triplicate, and immunoassay values were close to the lower limit of detection; therefore the <8% intra-assay and <10% interassay variation had no impact on the reproducibility of the results.

#### 2.4.4. High-Sensitivity C-Reactive Protein (hsCRP) Analysis

CRP levels in the study samples were analyzed at the Altamedica SpA Laboratory, Rome, Italy, by nephelometry using the Siemens N Cardio Phase hsCRP, 150 tests (Siemens Healthcare, Milan, Italy) and according to the manufacturer's instructions.

#### 2.4.5. Statistical Analysis

All values were expressed as mean and standard deviation. The comparison of averages was performed using the Pearson test for correlation between paired values. Missing data and values out of range were excluded from the analysis. The values were significant for *p* ≤ 0.001, and the changes were expressed by Δ%. The *R* coefficient of linear correlation applied to the dose-effect analysis was significant for *R*-squared >0.8. All the results were validated with the statistical program SPSS (v 20.0).

## 3. Results

**Figure 1:**
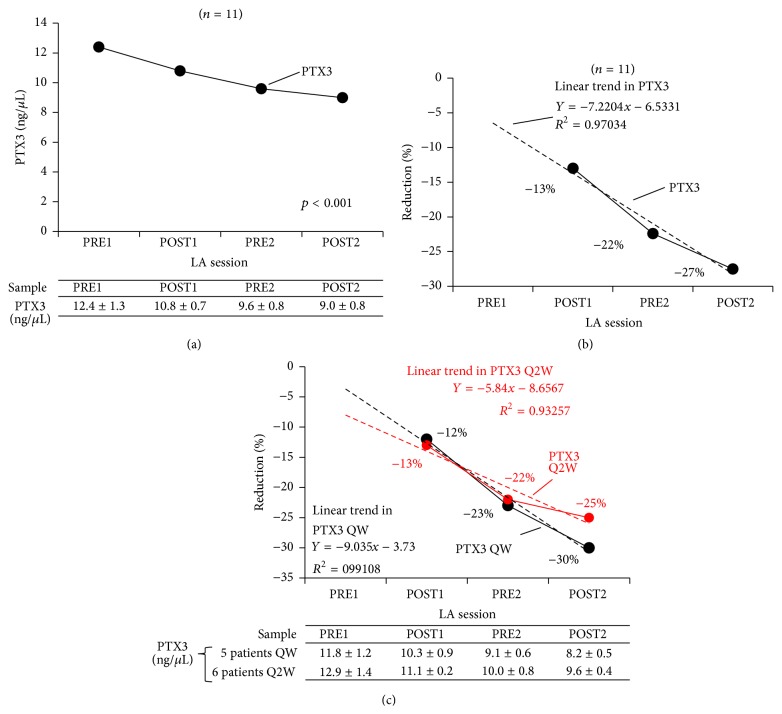
Effect of LA treatment on PTX3 mRNA expression (a), change in expression (b), and change in expression by LA schedule (c). LA, lipoprotein apheresis; PTX3, pentraxin 3; QW, once weekly; Q2W, biweekly; PRE1, blood sample collected before first LA session; POST1, blood sample collected after first LA session; PRE2, blood sample collected before second LA session; POST2, blood sample collected after second LA session.

A statistically significant downregulation of PTX3 mRNA expression was observed in all 11 patients (*p* < 0.001), as shown in Figure 1(a). The observed reduction started with the first LA session (from 12.4 ± 1.3 ng/*μ*L PRE1 to 10.8 ± 0.7 ng/*μ*L POST1) and was progressively enhanced during the interval between the first and second LA sessions (to 9.6 ± 0.8 ng/*μ*L at PRE2), probably due to modulation of the signal, and a maximum decrease was achieved after the second LA session (to 9.0 ± 0.8 ng/*μ*L POST2). A statistically significant treatment-effect linear correlation with *R* ^2^ of 0.97 (minimum −13%, maximum −27%) was observed for change in PTX3 mRNA expression, with progressive reduction as shown in Figure 1(b). The observed reduction in PTX3 mRNA expression was statistically significantly greater in patients undergoing weekly LA treatment sessions (*R* ^2^ = 0.99; minimum −12%, maximum −30%) than in patients undergoing treatment biweekly (*R* ^2^ = 0.93; minimum −13%, maximum 25%). The effect of LA treatment schedule on change in PTX3 mRNA expression is shown in Figure 1(c).

**Figure 2:**
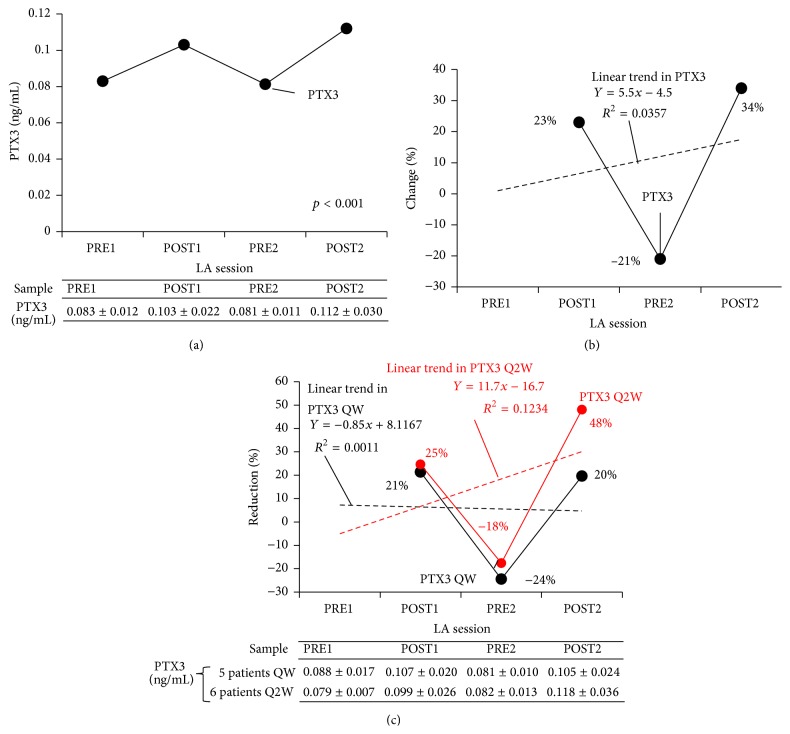
Effect of LA treatment on PTX3 peptide expression (ELISA) (a) and change in expression in all patients (b) and by LA schedule (c) in patients with HyperLp(a) (*n* = 11). LA, lipoprotein apheresis; PTX3, pentraxin; ELISA, enzyme-linked immunosorbent assay; HyperLp(a), hyperLp(a)lipoproteinemia; POST1, blood sample collected after first LA session; PRE2, blood sample collected before second LA session; POST2, blood sample collected after second LA session.

Plasma levels of soluble PTX3 peptide oscillated over the course of the two LA sessions. After a slight increase after the first LA session, from 0.08 ng/mL PRE1 to 0.1 ng/mL POST1, plasma soluble PTX3 levels (PTX peptide expression, assessed by ELISA) fell to 0.008 ng/mL PRE2 but climbed again after the second LA session (to 0.112 ng/mL POST2), as shown in Figure 2(a). Linear regression analysis revealed no statistically significant treatment-effect for LA on plasma soluble PTX3 levels (Figures 2(b) and 2(c)).

**Figure 3:**
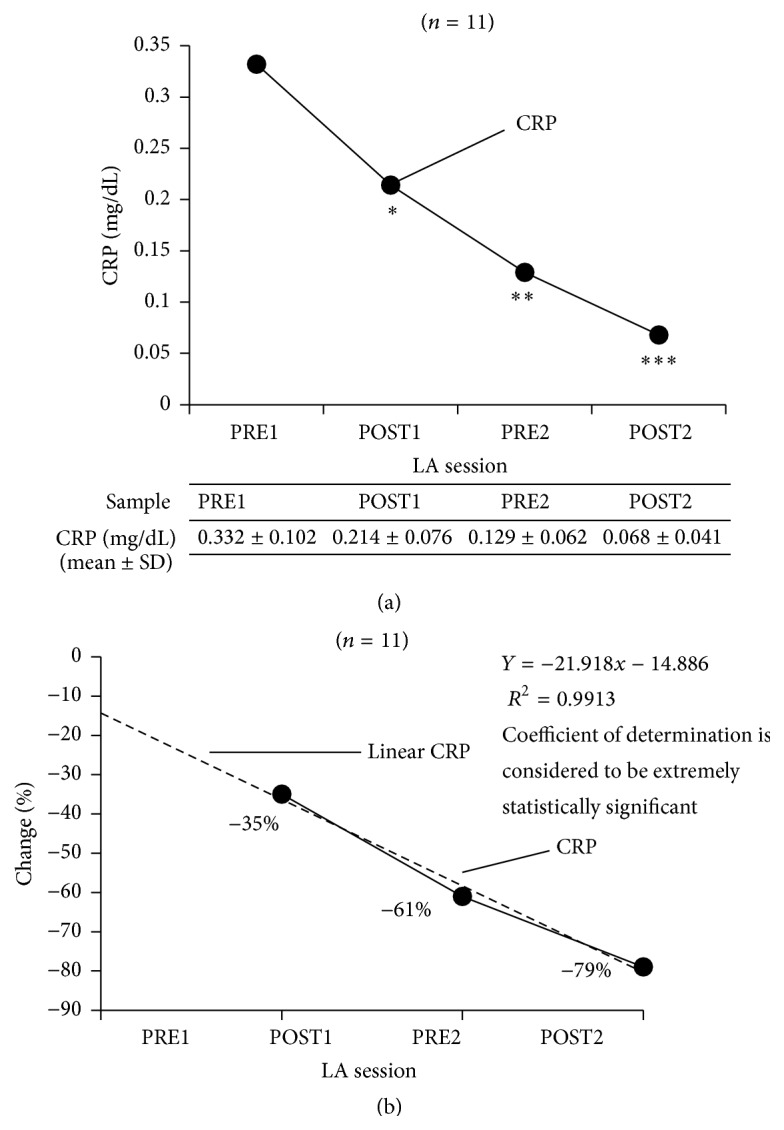
Effect of LA treatment on CRP expression (a), change in expression (b). ^*∗*^PRE1 versus POST1 two-tailed *p* value of 0.0039 (considered to be very statistically significant). ^*∗∗*^PRE1 versus PRE2 two-tailed *p* value <0.0001 (considered to be extremely statistically significant). ^*∗∗∗*^PRE2 versus POST2 two-tailed *p* value <0.0001 (considered to be extremely statistically significant). LA, lipoprotein apheresis; PTX3, pentraxin 3; QW, once weekly; Q2W, biweekly; PRE1, blood sample collected before first LA session; POST1, blood sample collected after first LA session; PRE2, blood sample collected before second LA session; POST2, blood sample collected after second LA session.

C-reactive protein (CRP) levels were also progressively reduced, starting from the first LA session (from 0.33 ± 0.10 mg/dL PRE1 to 0.21 ± 0.08 mg/dL POST1, 0.13 ± 0.06 mg/dL PRE2, and 0.07 ± 0.04 mg/dL POST2), and a statistically significant linear correlation for treatment-effect was observed for the change in plasma CRP levels (*R* ^2^ = 0.99; minimum −35%, maximum −79%), as shown in Figure 3.

## 4. Discussion

We studied the effects of a sustained reduction of apoB100-containing lipoproteins, achieved via LA with the DALI system, on the expression of mRNA which codifies PTX3 synthesis and on plasma levels of soluble PTX3 protein and CRP, in patients diagnosed with HyperLp(a) and AWA/CAD. The increase in plasma levels of soluble PTX3 observed at the first treatment, present in all patients in this study, including those treated every 7 days or every 15 days, might be due to an initial proinflammatory effect of the treatment. It has previously been noted that hemodialysis increases plasma levels of PTX3. A potential relationship between PTX3 and various cytokines removed during extracorporeal treatment might exist [12–15]. Moreover, evidence from literature has suggested a link between PTX3 and atherosclerosis. PTX3 is able to interfere with plaque stability due to its link to fibroblast growth factor 2. In fact, fibroblast growth factor 2 plays a key role in the induction, proliferation, migration, and survival of vascular smooth muscle cells and in the induction and excessive proliferation of smooth muscle cells in plaques. In addition foam cells are able to stimulate the expression of PTX3 in atherosclerotic plaques generating acute inflammation [16, 17]. Evidence also exists that PTX3 is implicated in the atherosclerotic process by means of other several mechanisms, including interaction with modified lipoproteins (oxidized low-density lipoprotein), formation of foam cells, and the activation of the fall in complement. In 2002, Rolph et al. highlighted the presence of PTX3 during a carotid thromboendarterectomy that was not present in healthy mammary arteries and thus assumed that PTX3 is involved in the proatherogenic inflammatory process [5]. PTX3 levels are elevated in patients with CVD [6, 16], and elevated levels in the general population are considered predictors of CVD, especially in patients with arterial hypertension affected by dyslipidemia [18, 19]. Zanetti et al. observed a direct correlation between hypertriglyceridemia and plasma PTX3 concentrations and an inverse correlation between HDL-cholesterol and PTX3 [19]. In 2000 it was reported that PTX3 increases during acute inflammation in patients suffering from acute myocardial infarction [6], and for this reason PTX3 is considered to be a new biochemical marker in acute myocardial infarction, along with troponin and creatine kinase [20]. Moreover, a study by Ryu and colleagues evaluated 376 patients who had ischemic stroke between September 2004 and September 2006. During follow-up, 19.4% of the patients died and these patients had the highest median PTX3 levels [21]. According to Ryu's study and other more recent studies, elevated PTX3 levels are independently associated with an increased mortality rate after ischemic ictus. This can mean that PTX3 can be used as a significant prognostic biomarker for patients with ischemic ictus [16, 21]. In addition, several different clinical studies have shown that PTX3 was associated with renal dysfunction and also, as has been noted, that it represents an important risk factor for predicting cardiovascular events [22, 23]. PTX3 is notably elevated in patients with terminal chronic kidney disease and the relationship between PTX3 and cardiovascular morbidity suggests a possible connection between PTX3 and arteriosclerosis and CVD in patients undergoing hemodialysis therapy [12].

In our study, PTX3 mRNA expression was elevated but soluble plasma protein levels were reduced by the apheresis sessions but rebounded after session. This suggests a different impact of DALI-LA on mRNA encoding PTX3 and the product of gene signaling action at cellular level. Other authors obtained a reduction of PTX3 with the HELP-LA system [24], which has a mechanism of action different to that of DALI-LA (i.e., precipitation induced by high dose of heparin for HELP versus filtration on gel of polyacrylate for DALI).

The apparent clearance of CRP by DALI in the absence of PTX3 clearance is worth noting. The two proteins have different molecular weight despite similar structural conformation. hsCRP is a member of the small pentraxins family with a molecular weight of 25,106 Da versus 40,165 Da for PTX3. The MW of LDL is much smaller at 514 kD. The pore size of the polyacrylate gel used for DALI might play a role in this effect, as could inflammatory effects caused by leachates from filters, tubing, and so forth. Certainly the picture at present is confusing, but the potential impacts on inflammatory process in the atherosclerotic plaque are intriguing.

C-reactive protein is an acute phase protein that appears in circulation in response to inflammatory cytokines, such as interleukin-6, and serves as a biomarker for systemic inflammation [25]. Measurement of CRP is useful for the detection and evaluation of infection, tissue injury, inflammatory disorders, and associated diseases, and it has been shown that CRP levels are of prognostic value in patients with myocardial infarction and unstable angina [26, 27]. High-sensitivity CRP (hsCRP) measurements are recognized as a strong independent risk marker for the identification of individuals at risk for future cardiovascular disease [28]. When used in conjunction with traditional clinical laboratory evaluation of acute coronary syndromes, measurements of hsCRP may be useful as an independent marker of prognosis for recurrent events in patients with stable coronary disease or acute coronary syndromes. In our study, we observed a progressive reduction in CRP levels from the first LA session and a statistically significant treatment-effect linear correlation for the change in plasma CRP levels.

In this study, LA (DALI system) showed essentially no effect on soluble PTX3 levels in plasma. However, importantly, it was associated with an intense downregulation of expression of PTX3 mRNA, which codifies PTX3 synthesis, suggesting a significant change/modulation at a posttranscriptional rather than posttranslational level that has not been previously reported in patients with severe genetically defined dyslipidemia and associated CAD. This is particularly evident because downregulation of PTX3 mRNA expression was not only linearly correlated with treatment (LA procedure), but also intensified if treatment was weekly, rather than biweekly. This may suggest that the observed downregulation is more intense when LA is more frequently applied. Thus, the conclusion of this study is that LA directly affects expression of mRNA codifying for PTX3, probably (but not definitely) by reducing apoB100-containing lipoproteins. In the light of the role of PTX3 as a proinflammatory and proatherogenic marker, it is possible that the application of sustained lipid-lowering treatment may induce antiatherogenic and (probably) anti-inflammatory effects that are linked not only to a reduction of apoB100-containing lipoproteins, but also to the direct removal of soluble PTX3 protein in plasma exerting a protective endothelial effect. In clinical practice, this might be achieved by LA treatment and also possibly by the most effective available cholesterol-lowering drugs (HMGCoA-reductase inhibitors/statins). The effects of the latest generation of lipid-lowering drugs, including proprotein convertase subtilisin/kexin type 9 inhibitors, cholesteryl ester transfer protein inhibitors, microsomal triglyceride transfer protein inhibitors, and apolipoprotein B synthesis inhibitors, should also be investigated. One last issue for consideration is that in the light of these findings, and from the evidence available from both short- and long-term studies, whether the inhibition of PTX3 achieved by sustained therapeutic lipid-lowering is transitory or stable should be investigated and confirmed. Thus, additional follow-up of patients with genetically determined dyslipidemia and CAD is required, with well-planned, continuous, long-term treatment to further investigate the therapeutic potential of lowering PTX3 directly or through gene-silencing treatments. In particular, a longitudinal study could offer further insights into cardiovascular hard endpoints, including acute myocardial infarction, stroke, and need for revascularization interventions.

In conclusion, LA via the DALI method reduced the expression of PTX3 at the level of mRNA, and this may contribute to the apparent anti-inflammatory effects of the procedure.

## Figures and Tables

**Table 1:** Patient demographics and diagnosis and details of lipoprotein apheresis treatment.

Patient (*N* = 11)	Age, years	Male (*n* = 10) Female (*n* = 1)	Treatment frequency	Processed volume, mL	DALI configuration	Diagnosis	CAD
1	47	Male	Weekly	10000	1250	HyperLp(a)	AWA
2	62	Male	Weekly	10000	1250	HyperLp(a)	Yes
3	52	Male	Biweekly	10000	1250	HyperLp(a)	Yes
4	43	Male	Biweekly	10000	1250	HyperLp(a)	Yes
5	49	Male	Biweekly	10000	1000	HyperLp(a)	Yes
6	52	Female	Biweekly	10000	1250	HyperLp(a)	Yes
7	50	Male	Weekly	10000	1250	HyperLp(a)	Yes
8	73	Male	Biweekly	10000	1000	HyperLp(a)	Yes
9	51	Male	Weekly	10000	1250	HyperLp(a)	Yes
10	63	Male	Weekly	10000	1250	HyperLp(a)	Yes
11	66	Male	Weekly	10000	1250	HyperLp(a)	Yes

DALI, Direct Adsorption of Lipids; HyperLp(a), hyperLp(a)lipoproteinemia; CAD, coronary artery disease; AWA, arterial wall atherosclerosis assessed by angiography but no CAD.

## References

[1] Tillet W. S., Francis T. Jr. (1930). Serological reactions in pneumonia with a non protein somatic fraction of pneumococcus. *The Journal of Experimental Medicine*.

[2] Lee G. W., Lee T. H., Vilcek J. (1993). TSG-14, a tumor necrosis factor- and IL-1-inducible protein, is a novel member of the pentaxin family of acute phase proteins. *The Journal of Immunology*.

[3] Alles V. V., Bottazzi B., Peri G., Golay J., Introna M., Mantovani A. (1994). Inducible expression of PTX3, a new member of the pentraxin family, in human mononuclear phagocytes. *Blood*.

[4] Klouche M., Peri G., Knabbe C. (2004). Modified atherogenic lipoproteins induce expression of pentraxin-3 by human vascular smooth muscle cells. *Atherosclerosis*.

[5] Rolph M. S., Zimmer S., Bottazzi B., Garlanda C., Mantovani A., Hansson G. K. (2002). Production of the long pentraxin PTX3 in advanced atherosclerotic plaques. *Arteriosclerosis, Thrombosis, and Vascular Biology*.

[6] Peri G., Introna M., Corradi D. (2000). PTX3, a prototypical long pentraxin, is an early indicator of acute myocardial infarction in humans. *Circulation*.

[7] Latini R., Maggioni A. P., Peri G. (2004). Prognostic significance of the long pentraxin PTX3 in acute myocardial infarction. *Circulation*.

[8] Kunes P., Holubcova Z., Kolackova M., Krejsek J. (2012). Pentraxin 3(PTX 3): an endogenous modulator of the inflammatory response. *Mediators of Inflammation*.

[9] Stefanutti C., Vivenzio A., Ferraro P. M., Morozzi C., Belotherkovsky D. (2011). Apheresis-inducible cytokine pattern change in severe, genetic dyslipidemias. *Cytokine*.

[10] Thompson G. R. (2013). The evidence-base for the efficacy of lipoprotein apheresis in combating cardiovascular disease. *Atherosclerosis Supplements*.

[11] Stefanutti C., Julius U. (2013). Lipoprotein apheresis: state of the art and novelties. *Atherosclerosis Supplements*.

[12] Boehme M., Kaehne F., Kuehne A. (2007). Pentraxin 3 is elevated in haemodialysis patients and is associated with cardiovascular disease. *Nephrology Dialysis Transplantation*.

[13] Stefanutti C., Morozzi C., Petta A. (2011). Lipid and low-density-lipoprotein apheresis. Effects on plasma inflammatory profile and on cytokine pattern in patients with severe dyslipidemia. *Cytokine*.

[14] Zhou Y., Ni Z., Zhang J. (2013). Plasma pentraxin 3 may be a better marker of peripheral artery disease in hemodialysis patients than C-reactive protein. *Vascular Medicine*.

[15] Oleśkowska-Florek W., Połubinska A., Baum E. (2014). Hemodialysis-induced changes in the blood composition affect function of the endothelium. *Hemodialysis International*.

[16] Mantovani A., Garlanda C., Bottazzi B. (2006). The long pentraxin PTX3 in vascular pathology. *Vascular Pharmacology*.

[17] Jylhävä J., Haarala A., Kähönen M. (2011). Pentraxin 3 (PTX3) is associated with cardiovascular risk factors: the Health 2000 Survey. *Clinical and Experimental Immunology*.

[18] Bassi N., Zampieri S., Ghirardello A. (2009). Pentraxins, anti-pentraxin antibodies, and atherosclerosis. *Clinical Reviews in Allergy & Immunology*.

[19] Zanetti M., Bosutti A., Ferreira C. (2009). Circulating pentraxin 3 levels are higher in metabolic syndrome with subclinical atherosclerosis: evidence for association with atherogenic lipid profile. *Clinical and Experimental Medicine*.

[20] Ristagno G., Santonocito C., Li Y., Volti G. L., Gullo A. (2010). Biomarkers of myocardial injury after cardiac arrest or myocardial ischemia. *Frontiers in Bioscience (Scholar Edition)*.

[21] Ryu W.-S., Kim C. K., Kim B. J., Kim C., Lee S.-H., Yoon B.-W. (2012). Pentraxin 3: a novel and independent prognostic marker in ischemic stroke. *Atherosclerosis*.

[22] Kunes P., Mandak J., Holubcova Z., Kolackova M., Krejsek J. (2013). The long pentraxin PTX3: a candidate anti-inflammatory mediator in cardiac surgery. *Perfusion*.

[23] Speeckaert M. M., Speeckaert R., Carrero J. J., Vanholder R., Delanghe J. R. (2013). Biology of human pentraxin 3 (PTX3) in acute and chronic kidney disease. *Journal of Clinical Immunology*.

[24] Zanetti M., Zenti M., Barazzoni R. (2014). HELP LDL apheresis reduces plasma pentraxin 3 in familial hypercholesterolemia. *PLoS ONE*.

[25] Pearson T. A., Mensah G. A., Alexander R. W. (2003). Markers of inflammation and cardiovascular disease. Application to clinical and public health practice. A statement for healthcare professionals from the centers for disease control and prevention and the American Heart Association. *Circulation*.

[26] Dedobbeleer C., Melot C., Renard M. (2004). C-reactive protein increase in acute myocardial infarction. *Acta Cardiologica*.

[27] Heeschen C., Hamm C. W., Bruemmer J., Simoons M. L. (2000). Predictive value of C-reactive protein and troponin T in patients with unstable angina: a comparative analysis. *Journal of the American College of Cardiology*.

[28] Ridker P. M. (2001). High-sensitivity C-reactive protein. Potential adjunct for global risk assessment in the primary prevention of cardiovascular disease. *Circulation*.

